# Tumor Size Still Impacts Prognosis in Breast Cancer With Extensive Nodal Involvement

**DOI:** 10.3389/fonc.2021.585613

**Published:** 2021-04-09

**Authors:** Yin Liu, Min He, Wen-Jia Zuo, Shuang Hao, Zhong-Hua Wang, Zhi-Ming Shao

**Affiliations:** ^1^ Department of Breast Surgery, Fudan University Shanghai Cancer Center/Cancer Institute, Shanghai, China; ^2^ Department of Oncology, Shanghai Medical College, Fudan University, Shanghai, China; ^3^ Institutes of Biomedical Science, Fudan University, Shanghai, China

**Keywords:** tumor size, extensive nodal involvement, breast cancer, prognosis, staging

## Abstract

**Background and Purpose:**

Although tumor size and nodal status are the most important prognostic factors, it is believed that nodal status outperforms tumor size as a prognostic factor. In particular, when patients have a nodal stage greater than N2 (more than nine positive lymph nodes), it is well accepted that tumor size does not retain its prognostic value. Even in the newest American Joint Committee on Cancer (AJCC) prognostic staging system, which includes molecular subtype as an important prognostic factor, T1-3N2 patients are categorized as the same population. The same is true for T1-4N3 patients. Moreover, some physicians have speculated that for tumors staged N2 or greater, the smaller the tumor is, the more aggressive the tumor. Thus, this study aims to investigate the prognostic value of tumor stage (T stage) in patients with extensive nodal involvement and to compare the survival of T4N × M0 and T × N3M0.

**Patients and Methods:**

Female breast cancer patients with nine or more positive lymph nodes or with T4 tumors were identified in the SEER registry between 2010 and 2015. The effect of T stage on breast cancer-specific survival (BCSS) was assessed using the Kaplan–Meier survival curve method and risk-adjusted Cox proportional hazard regression modeling. Survival comparison of T4NxM0 and TxN3M0 patients was also achieved using the Kaplan–Meier survival curve method and risk-adjusted Cox proportional hazard regression model.

**Results:**

Overall, 21,696 women with N2-3 tumors were included from 284,073 patients.T stage, nodal stage (N stage), ER, PR, HER2 and grade were all independent prognostic factors (p <0.001). HRs for ER, PR, HER2, grade, and N stage were 0.662 (0.595–0.738), 0.488 (0.438–0.543), 0.541 (0.489–0.598), 1.534 (1.293–1.418) and 1.551 (1.435–1.676), respectively. Notably, HER2 positivity was correlated with better BCSS possibly due to the wide adoption of anti-HER2 therapy. Using T1 as a reference, HRs of T2, T3, and T4 were 1.363 (1.200–1.548), 2.092 (1.824–2.399) and 3.497 (3.045–4.017), respectively. The same results held true when subgroup analysis based on N stage were conducted. In the two subgroups, namely, women staged as T1-3N2 and women staged as T1-4N3, T stage was also a significant negative prognostic factor independent of ER, PR, HER2 and grade. Moreover, 8,328 women staged as T4 with different nodal statuses were also identified from the whole database. When we compared T4Nx with TxN3, it was found that T4 tumors exhibited worse outcomes than N3 tumors independent of other prognostic factors. When molecular subtype was included in the subgroup analysis, survival could not be distinguished between T4 and N3 only in TNBC.

**Conclusions:**

In patients with extensive nodal status, tumor stage remains a prognostic factor independent of other factors, such as ER, PR, HER2, and grade. In patients with T4Nx or TxN3 tumors, T4 tumors exhibit worse outcomes than N3 tumors independent of other prognostic factors. The AJCC staging system should be modified based on these findings.

## Background and Purpose

Several classical and well-accepted prognostic factors have been reported in breast cancer, including tumor size, axillary lymph node (LN) status, histologic grade, hormone receptor status, HER2/neu status, and the presence of lymphovascular invasion (LVI) (1–5). Among these factors, axillary LN status and tumor size are two of the most important factors included in the American Joint Committee on Cancer (AJCC) staging system (6). Due to screening and early diagnosis, breast cancer patients are diagnosed at earlier stages (7). Nevertheless, 10–15% of breast cancer patients have been diagnosed with extensive nodal involvement in the US (8), and the situation in developing countries is even less optimistic (9). Although tumor size and axillary LN status are the most important prognostic factors, it is believed that LN nodal status outperforms tumor size as a prognostic factor. In particular, when patients have a nodal stage greater than N2 (more than nine positive lymph nodes), it is well accepted that tumor size does not retain its prognostic value according to the AJCC staging system (6, 10). Even in the newest AJCC prognostic staging system, which includes molecular subtype as an important prognostic factor, T1-3N2 patients are categorized as the same population as that in the traditional AJCC pathological staging system. The same is also true for T1-4N3 tumors. Moreover, some physicians speculated that for N2 or greater staged tumors, the smaller the tumor is, the more aggressive the tumor (8). Thus, this study aims to investigate the prognostic value of tumor stage (T stage) in patients with extensive nodal involvement and to compare the survival of T4NxM0 (categorized as IIIB in the AJCC staging system) and TxN3M0 (categorized as IIIC in the AJCC staging system) patients.

## Patients and Methods

### Data Source

We abstracted data from the Surveillance, Epidemiology, and End Results (SEER) 18 Registries Research Data + Hurricane Katrina Impacted Louisiana Cases. In total, this database covers approximately 28% of the total US population. The data reported in this study represent the recent follow-up data (December 31, 2015) available in the SEER database (11).

### Cohort Selection

We used SEER*Stat version 8.3.5 to generate a case list. We extracted cases of female breast cancer diagnosed from 2010 to 2015 because HER2 status was included in these data. We selected women who had a diagnosis of histologically confirmed first invasive breast cancer without distant metastasis. We generated a case listing with information on the following variables: year of diagnosis, age at diagnosis, AJCC pathological stage, AJCC tumor stage and size, AJCC nodal stage, estrogen receptor (ER) and progesterone receptor (PR) status, ERBB2 status (formerly HER2 or HER2/neu), cause of death and survival (months). We used the cause of death to site recode variables in SEER 18 to extract patient status at the time of the last follow-up. Based on this information, we grouped all patients into categories of alive or dead due to other causes and dead due to breast cancer. We used the survival time (months) variable to extract information on time from date of diagnosis to last follow-up. SEER*Stat estimates survival time in months by subtracting the date of diagnosis from the date of last contact (the study cutoff). The study cutoff date was December 31, 2015, which is the date of the last update on the follow-up time.

### Statistical Analyses

Descriptive statistics were used to examine the following baseline characteristics of breast cancer cases: year of diagnosis, age at diagnosis, American Joint Committee on Cancer (AJCC) pathological stage, AJCC tumor stage and tumor size, AJCC nodal stage, estrogen receptor (ER) and progesterone receptor (PR) status, ERBB2 status, cause of death and survival (months). To create a subcohort of women for whom the positivity or negativity of all three receptors (ER, PR, and ERBB2) was known, we excluded women whose ER and PR status were coded as borderline, undetermined whether positive or negative, or unknown. We further excluded women whose ERBB2 status was coded as borderline, equivocal, indeterminate, undetermined whether positive or negative, or unknown. We classified breast cancers into four groups: HR positive/ERBB2 negative, HR positive/ERBB2 positive, HR negative/ERBB2 positive and HR negative/ERBB2 negative. BCSS (breast cancer-specific survival) was used as the primary study outcome of the SEER data, which was calculated from the date of diagnosis to the date of breast cancer-specific death. The causes of death were categorized as either breast cancer related or non-breast cancer related. Patients who died of non-breast cancer related causes were censored according to the date of death. The effect of T stage on BCSS was assessed using the Kaplan–Meier survival curve method and risk-adjusted Cox proportional hazard regression modeling. The survival comparison of T4NxM0 and TxN3M0 were also conducted using the Kaplan–Meier survival curve method. We computed 95% confidence intervals for all point estimates (ORs and HRs). A P-value of 0.05 or less was considered statistically significant. All P-values were 2-tailed. All statistical analyses were performed using IBM SPSS Statistics 22.

## Results

### Multivariate Survival Analyses in N2-3 Patients

**Figure 1:**
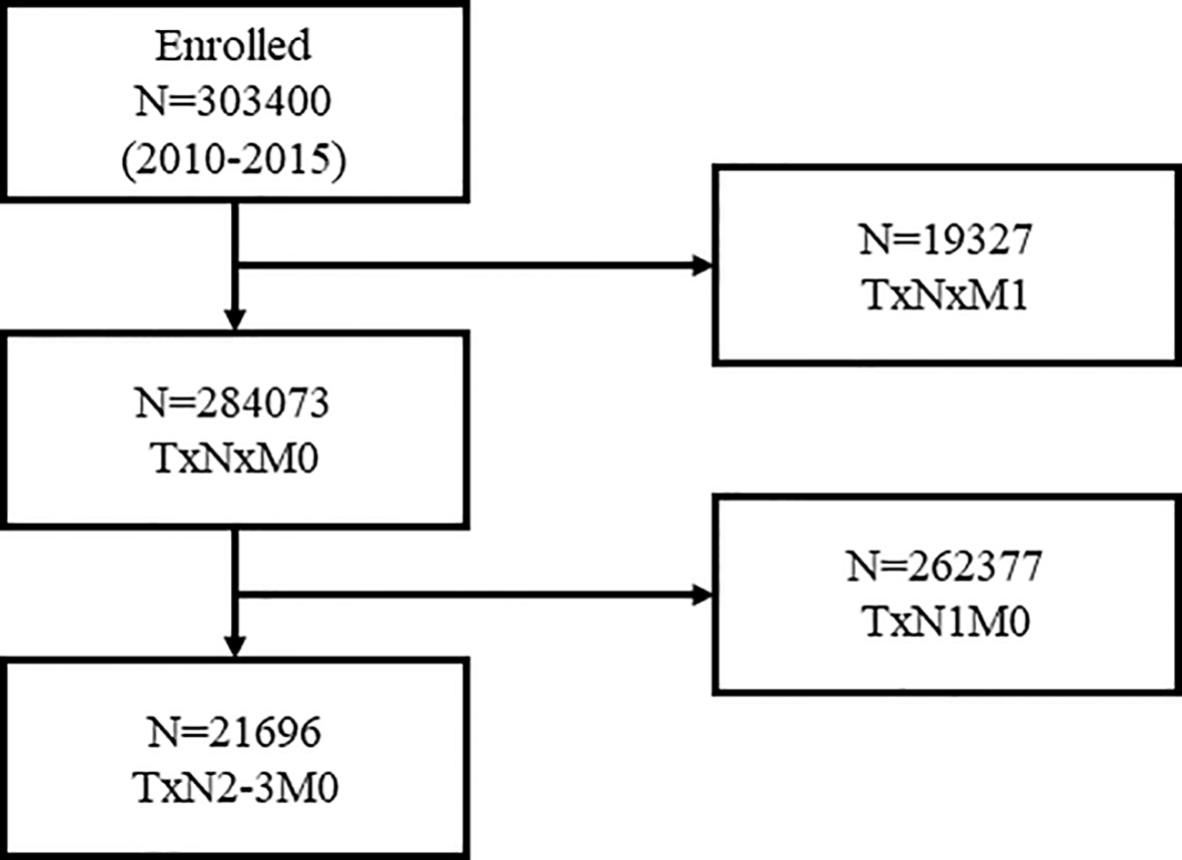
Patient flow chart for inclusion and exclusion.

**Figure 2:**
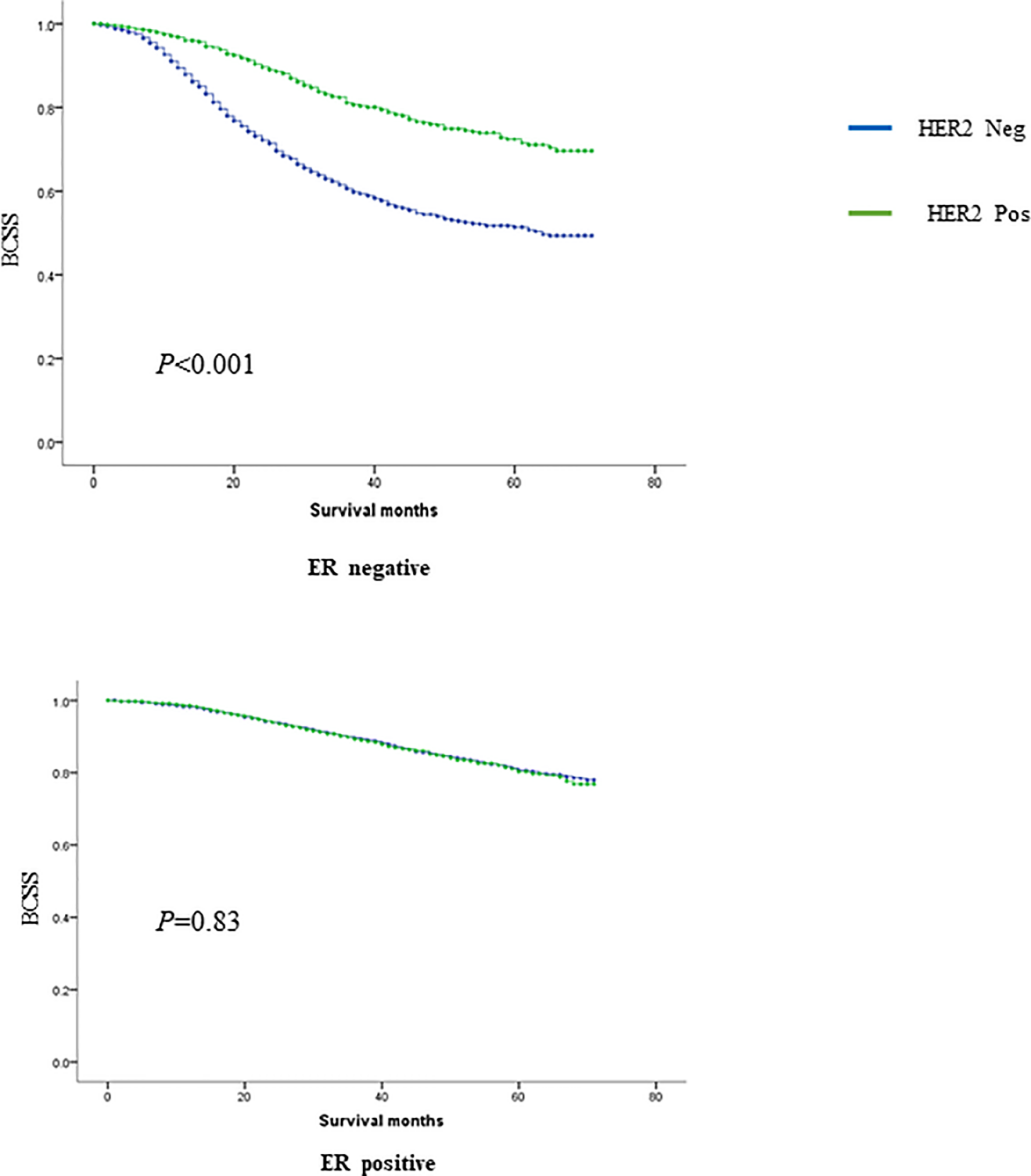
The prognostic value of HER2 in ER negative **(A)** and positive **(B)** tumors.

Overall, 21,696 women with N2-3 tumors were included from 284,073 patients (**Figure 1**). **Table 1** summarizes the sample demographics according to N stage. T stage, nodal stage (N stage), ER, PR, HER2 and grade were all independent prognostic factors (p <0.001). HRs of ER, PR, HER2, grade, and N stage were 0.662 (0.595–0.738), 0.488 (0.438–0.543), 0.541 (0.489–0.598), 1.534 (1.293–1.418) and 1.551 (1.435–1.676), respectively (**Table 2**). Notably, HER2 positivity was correlated with improved BCSS, possibly due to the wide adoption of anti-HER2 therapy. To further explore the prognostic value of HER2 status, we performed subgroup survival analyses according to ER status. Moreover, we found that HER2-positive status correlated with better outcome in ER negative patients but lost its prognostic value in ER-positive patients (**Figure 2**).

**Table 1:** Baseline characteristics according to N stage in N2–3 patients.

Characteristics	Total (n = 21696) n (%)	N2 (n = 13,997) n (%)	N3 (n = 7,699) n (%)	*P* value^a^
Race				.299
White	16,342 (75.3)	10,494 (75.0)	5,848 (76.0)	
Black	3,184 (14.7)	2,078 (14.8)	1,106 (14.3)	
Other^b^	2,039 (9.4)	1,337 (9.6)	702 (9.1)	
Unknown	131 (0.6)	88 (0.6)	43 (0.6)	
T stage				<0.001
T1	4,082 (18.8)	2,963 (21.2)	1,119 (14.5)	
T2	10,255 (47.3)	6,940 (49.6)	3,315 (43.1)	
T3	4,485 (20.7)	2,520 (18.0)	1,965 (25.5)	
T4	2,577 (11.9)	1,401 (10.0)	1,176 (15.3)	
Unknown	297 (1.3)	173 (1.2)	124 (1.6)	
Grade				<0.001
I-II	10,143 (46.8)	6,875 (49.1)	3,268 (42.4)	
III	10,431 (48.1)	6,481 (46.3)	3,950 (51.3)	
Unknown	1,122 (5.1)	641 (4.6)	481 (6.3)	
ER status				<0.001
Positive	16,470 (75.9)	1,0911 (78.0)	5,559 (72.2)	
Negative	4,860 (22.4)	2,850 (20.4)	2,010 (26.1)	
Unknown	366 (1.7)	236 (1.6)	130 (1.7)	
PR status				<0.001
Positive	13,799 (63.6)	9,239 (66.0)	4,560 (59.2)	
Negative	7,459 (34.4)	4,477 (32.0)	2,982 (38.7)	
Unknown	438 (2.0)	281 (2.0)	157 (2.1)	
HER2 status^c^				<0.001
Amplification	4,414 (20.3)	2,729 (19.5)	1,685 (21.9)	
Not amplification	16,289 (75.1)	10,620 (75.9)	5,669 (73.6)	
Unknown	993 (4.6)	648 (4.6)	345 (4.5)	
Subtype				<0.001
ER+HER2+	13,303 (61.3)	8,843 (63.2)	4,460 (57.9)	
ER+HER2-	2,877 (13.3)	1,850 (13.2)	1,027 (13.3)	
ER-HER2+	1,529 (7.0)	876 (6.3)	653 (8.5)	
ER-HER2-	2,965 (13.7)	1,764 (12.6)	1,201 (15.6)	
Unknown	1,022 (4.7)	664 (4.7)	358 (4.6)	
Cause of death				<0.001
Alive or Other^d^	18,690 (86.1)	12,454 (89.0)	6,236 (81.0)	
Breast Cancer	3,006 (13.9)	1,543 (11.0)	1,463 (19.0)	

ER, estrogen receptor; HER2, human epidermal growth factor receptor 2; PR, progesterone receptor.

a p value of the χ2 test comparing the N2 and N3 groups.

b Including American Indian/Alaskan native, and Asian/Pacific Islanders.

c HER2 amplification was defined as 3+ immunohistochemistry or gene amplification in fluorescence in situ hybridization.

d Other cause of death except breast cancer.

**Table 2:** Multivariate survival analysis by Cox proportional hazard regression modeling in TxN2-3M0 breast cancer patients.

	*P* value	HR	95%CI	
			Lower limit	Upper limit
T	<0.001			
T2 vs. T1	<0.001	1.363	1.200	1.548
T3 vs. T1	<0.001	2.092	1.824	2.399
T4 vs. T1	<0.001	3.497	3.045	4.017
N (N3 vs. N2)	<0.001	1.551	1.435	1.676
Grade (G3 vs. G1–2)	<0.001	1.354	1.293	1.418
ER (pos. vs. neg.)	<0.001	.662	.595	.738
PR (pos. vs. neg.)	<0.001	.488	.438	.543
HER2 (pos. vs neg.)	<0.001	.541	.489	.598

HR, Hazard Ratio; CI, Confidence Interval; pos, positive; neg, negative.

### Tumor Size Still Impacts Prognosis in Breast Cancer With Extensive Nodal Involvement

When T1 is used as a reference, HR values of T2, T3, and T4 were 1.363 (1.200–1.548), 2.092 (1.824–2.399) and 3.497 (3.045–4.017), respectively (**Table 1**). The same results held true when subgroup analysis was performed according to N stage. In the two subgroups of T1-3N2 and T1-4N3 women, T stage was also a significant negative prognostic factor independent of ER, PR, HER2, and grade. In T1-3N2 tumors, when we used T1 as reference, HR values of T2 and T3 were 1.899 (1.269–2.812) and 2.593 (1.661–4.047), respectively. In T1-4N3 women, when we use T1 as reference, HR values of T2, T3, and T4 were 1.146 (0.955–1.375), 1.803 (1.494–2.174) and 2.776 (2.293–3.359), respectively (**Table 3**).

**Table 3:** Multivariate survival analysis by Cox proportional hazard regression modeling in T1–3N2M0 and T1–4N3M0 breast cancer patients.

	T1–3N2M0				T1–4N3M0			
	*P* value	HR	95% CI		*P* value	HR	95% CI	
			Lower limit	Upper limit			Lower limit	Upper limit
T	<0.001				<0.001			
T2 vs. T1	<0.001	1.899	1.269	2.812	.144	1.146	.955	1.375
T3 vs. T1	<0.001	2.593	1.661	4.047	<0.001	1.803	1.494	2.174
T4 vs. T1	N/A	N/A	N/A	N/A	<0.001	2.776	2.293	3.359
Grade (G3 vs. G1–2)	<0.001	1.381	1.046	1.693	<0.001	1.348	1.163	1.594
ER (pos. vs. neg.)	.773	.943	.674	1.320	<0.001	.534	.461	.620
PR (pos. vs. neg.)	<0.001	.507	.357	.721	<0.001	.599	.516	.695
HER2 (pos. vs. neg.)	<0.001	.612	.424	.793	<0.001	.542	.435	.637

### T4NxM0 Indicates Worse Prognosis Than TxN3M0

**Figure 3:**
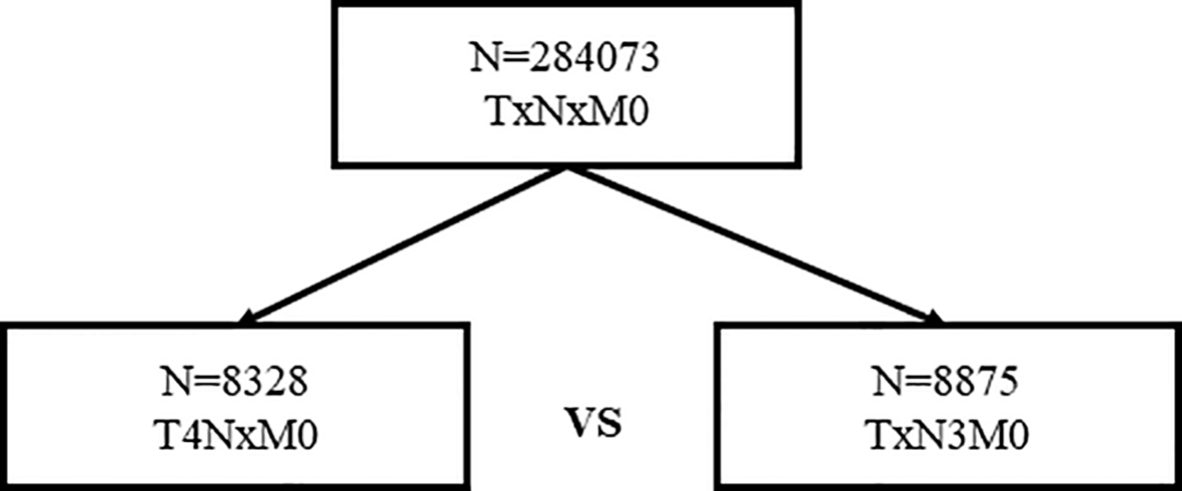
Patient selection for survival comparison of T4 and N3 tumors.

**Figure 4:**
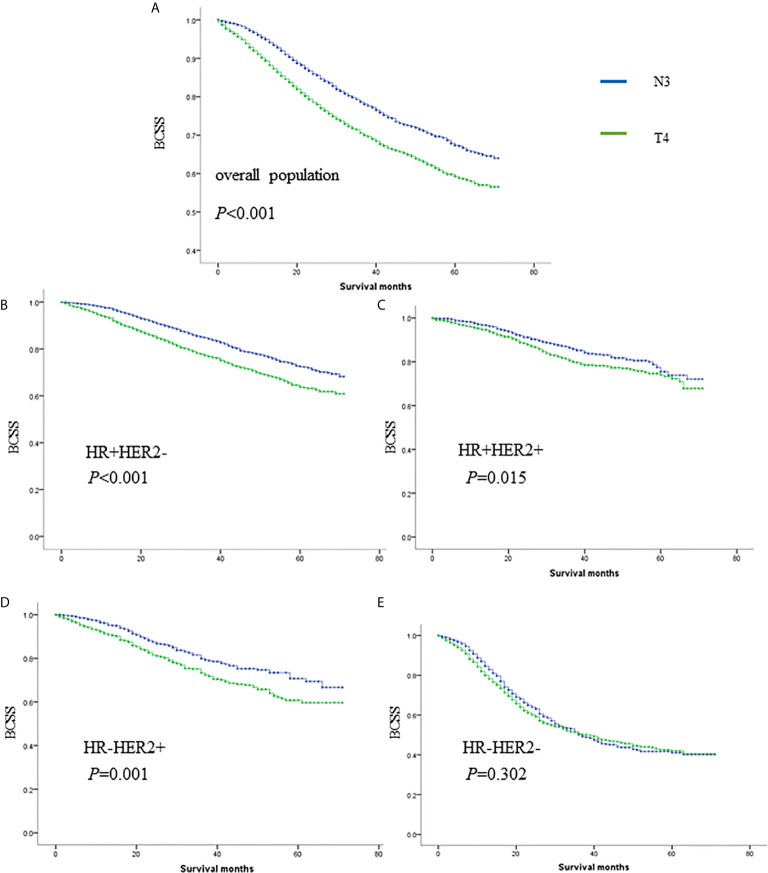
The survival comparisons of T4 and N3 tumors in whole population **(A)**, HR+HER2- **(B)**, HR+HER2+ **(C)**, HR-HER2+ **(D)** and HR-HER2- **(E)** subgroups.

In total, 8,328 women staged as T4 with different nodal statuses were also identified from the whole database (**Figure 3**). T4N1-2 patients are categorized as IIIB in the AJCC pathological staging system, while T1-4N3 patients are categorized as IIIC. However, when we compared T4Nx with TxN3 tumors in this SEER database, T4 tumors had worse outcomes than N3 tumors independent of other prognostic factors (**Figure 4A**). When ER and HER2 status were included in subgroup analysis, we found that T4 indicated worse prognosis than N3 if tumors were ER or HER2 positive (**Figures 4B–D**). Only in ER-HER2− patients (mostly triple negative tumors), T4 exhibited a similar prognosis as N3 (**Figure 4E**). Given that the malignant degree of ER-HER2- breast cancer is too high, it is possible that the influence of traditional T and N stage will be relatively small.

## Discussion

Breast cancer staging is a classical and even outdated proposition. The traditional tumor stage is becoming less important with the development of molecular subtyping and precision treatment. The latest version of the AJCC prognostic staging system is a fusion of molecular subtypes and traditional pathological indicators. However, in recent years, some studies on T stage and N stage have been performed to explore the correlation between these two factors and their influence on prognosis. Yu et al. found that in LN-negative diseases, the relationship between tumor size and breast cancer-specific mortality (BCSM) was piecewise. Using 21- to 30-mm tumors as the reference, the HR of BCSM increased with increasing tumor size until it reached a peak at 41 to 50 mm, after which increasing tumor size was unexpectedly related to reduced hazard ratios, with a nadir at 61 to 80 mm (12). More interestingly, Jennifer Y. Wo’s work indicated that small tumors with four positive LNs might predict higher BCSM compared with larger tumors. In extensive node-positive disease, very small tumor size may be a surrogate for biologically aggressive disease (8). Therefore, in the field of traditional staging, some controversial results are noted. In the AJCC staging system, the prognostic value of T staging is ignored when nodal staging is higher. For instance, T1-3N2 patients are categorized as the same population. The same is true for T1-4N3 patients. However, is that truly the case? Does tumor size truly lose its prognostic value when lymph nodes are extensively involved? After exploring a large database, we found that it was not the case. In our study, we found that in T1-3N2 and T1-4N3 women, T stage was still a significant prognostic factor independent of ER/PR/HER2/grade. To better illustrate the prognostic value of T stage, 8,328 women staged as T4 with different nodal statuses were also identified from the whole database. When we compared T4Nx with TxN3, T4 tumors had worse outcomes than N3 tumors independent of other prognostic factors. However, T4 was classified as IIIB, while N3 was classified as IIIC in the 8th AJCC staging system. The underlying mechanism of our outcome might also be explained as follows. It has been proposed that a higher fixed proportion of cancer cells with stem-cell properties of self-renewing and pluripotency, which we refer to as cancer stem cells (13), may be present, and these cells may allow small tumors to metastasize to distant sites. However, the degree of malignancy of cancer stem cells is determined by two factors: migration ability and proliferation ability. Tumors with extensive lymph node involvement when they are small tend to have strong migration ability but not proliferation ability, whereas large tumors with extensive lymph node involvement tend to have strong migration and proliferation abilities.

To the best of our knowledge, this is the largest and most recent study evaluating the prognostic significance of tumor size in extensive node-positive breast cancer, and the results pose a challenge to the AJCC staging system. However, there are still limitations that should be mentioned. First, this is a retrospective study with inevitable bias. Additionally, the SEER database does not contain treatment information, so the effect of treatment on prognosis cannot be excluded.

In summary, in patients with extensive nodal status, tumor stage remains a prognostic factor independent of other factors, such as ER, PR, HER2, and grade. In patients with T4Nx or TxN3 tumors, T4 tumors have worse outcomes than N3 tumors independent of other prognostic factors. The AJCC staging system should be slightly modified based on these outcomes.

## Data Availability Statement

Publicly available datasets were analyzed in this study. This data can be found here: Surveillance, Epidemiology, and End Results (SEER) database (https://seer.cancer.gov/).

## Ethics Statement

Ethical review and approval was not required for the study on human participants in accordance with the local legislation and institutional requirements. Written informed consent for participation was not required for this study in accordance with the national legislation and the institutional requirements.

## Author Contributions

YL and SH collected the data. YL and MH analyzed the data. YL wrote the original manuscript. YL, W-JZ, Z-HW and Z-MS reviewed and edited the manuscript. Z-HW and Z-MS supervised the study. All authors contributed to the article and approved the submitted version.

## Conflict of Interest

The authors declare that the research was conducted in the absence of any commercial or financial relationships that could be construed as a potential conflict of interest.
